# A custom-made guide-wire positioning device for Hip Surface Replacement Arthroplasty: description and first results

**DOI:** 10.1186/1471-2474-11-161

**Published:** 2010-07-14

**Authors:** Martijn Raaijmaakers, Frederik Gelaude, Karla De Smedt, Tim Clijmans, Jeroen Dille, Michiel Mulier

**Affiliations:** 1 Department of Reconstructive Hip Surgery, UZ Pellenberg, Katholieke Universiteit Leuven, Belgium; 2 Materialise NV, Leuven, Belgium

## Abstract

**Background:**

Hip surface replacement arthroplasty (SRA) can be an alternative for total hip arthroplasty. The short and long-term outcome of hip surface replacement arthroplasty mainly relies on the optimal size and position of the femoral component. This can be defined before surgery with pre-operative templating. Reproducing the optimal, templated femoral implant position during surgery relies on guide wire positioning devices in combination with visual inspection and experience of the surgeon. Another method of transferring the templated position into surgery is by navigation or Computer Assisted Surgery (CAS). Though CAS is documented to increase accurate placement particularly in case of normal hip anatomy, it requires bulky equipment that is not readily available in each centre.

**Methods:**

A custom made neck jig device is presented as well as the results of a pilot study.

The device is produced based on data pre-operatively acquired with CT-scan. The position of the guide wire is chosen as the anatomical axis of the femoral neck. Adjustments to the design of the jig are made based on the orthopedic surgeon's recommendations for the drill direction. The SRA jig is designed as a slightly more-than-hemispherical cage to fit the anterior part of the femoral head. The cage is connected to an anterior neck support. Four knifes are attached on the central arch of the cage. A drill guide cylinder is attached to the cage, thus allowing guide wire positioning as pre-operatively planned.

Custom made devices were tested in 5 patients scheduled for total hip arthroplasty. The orthopedic surgeons reported the practical aspects of the use of the neck-jig device. The retrieved femoral heads were analyzed to assess the achieved drill place in mm deviation from the predefined location and orientation compared to the predefined orientation.

**Results:**

The orthopedic surgeons rated the passive stability, full contact with neck portion of the jig and knife contact with femoral head, positive. There were no guide failures. The jig unique position and the number of steps required to put the guide in place were rated 1, while the complexity to put the guide into place was rated 1-2. In all five cases the guide wire was accurately positioned. Maximum angular deviation was 2.9° and maximum distance between insertion points was 2.1 mm.

**Conclusions:**

Pilot testing of a custom made jig for use during SRA indicated that the device was (1) successfully applied and user friendly and (2) allowed for accurate guide wire placement according to the preoperative plan.

## Background

Hip surface replacement arthroplasty (SRA) can be an alternative for total hip arthroplasty. With good patient selection it offers several benefits compared to conventional total hip arthroplasty (THA) [1-3]. Due to the larger head diameter, SRA has a better implant stability and a decreased risk for dislocation [1,4]. Increased inherent implant stability in turn decreases the need to lengthen the femur or to increase offset for soft tissue tensioning [5] resulting in less leg length discrepancy and preventing excessive offset. Furthermore, the proximal femoral bone stock is preserved with SRA [6] making it possible to use a standard THA femoral component if revision of the femoral component would be necessary. And compared to THA, SRA can better approximate normal hip kinematics [7].

Although SRA has good short and medium term results in young and active patients [8,9], it has specific potential complications. The most frequent encountered complication is fracture of the femoral neck followed by aseptic loosening of the femoral component. Both these complications increase with less accurate positioning of the femoral implant [10-13].

The optimal size and position of the femoral component can be defined before surgery with pre-operative templating. Reproducing the optimal, templated femoral implant position during surgery relies on guide wire positioning devices in combination with visual inspection and experience of the surgeon. Another method of transferring the templated position into surgery is by navigation or Computer Assisted Surgery (CAS). Because the latter method requires bulky instrumentation a smaller tool that provides comparable accuracy would be a valuable addition to the instrumentarium of the orthopedic surgeon. We developed a custom made neck jig that can be used to guide the femoral component of SRA, possibly as an alternative to CAS. This study describes an in-vivo study on the practical usability of the jig, and ex-vivo assessment of the accuracy of the guide wire placement.

## Methods

### The neck jig design

The neck jig as method for transferring the templated position of the guide wire into surgery was evaluated in five patients scheduled for total hip arthroplasty. The neck jig is custom designed for each individual patient. Pre-operative CT scans in slices of 2.5 mm are made from the femoral head until 5 centimeters under the level of the lesser trochanter. The scan data are converted into DICOM format and as such imported into the Medical Image Processing software Mimics^® ^(Materialise NV, Leuven, Belgium).

**Figure 1:**
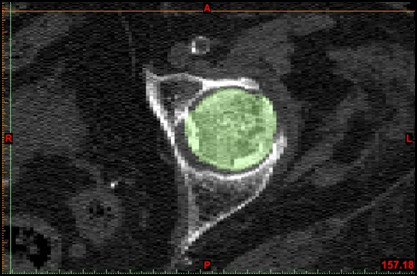
**Medical Image Processing**. Segmentation of bony tissue. (axial CT image of proximal left femur; bony tissue highlighted in color). [Mimics^© ^screenshot].

**Figure 2:**
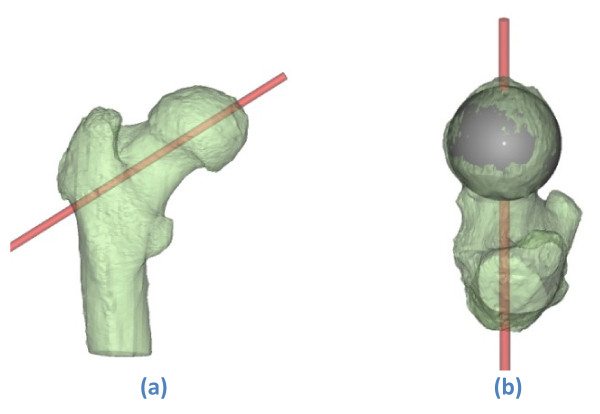
**Planned guide wire direction on a (transparently visualised) 3 D model of the proximal femur**. (a)-(b) Seen from posterior and superior respectively. [Mimics^© ^screenshot].

Femoral three dimensional bone surface models are extracted from the CT images (Figure 1) using the optimal parameters settings as defined by Gelaude et al [14]. The position of the guide wire is chosen as the anatomical axis of the femoral neck. Adjustments to the design of the jig are made based on the orthopedic surgeon's recommendations for the drill direction (Figure 2).

**Figure 3:**
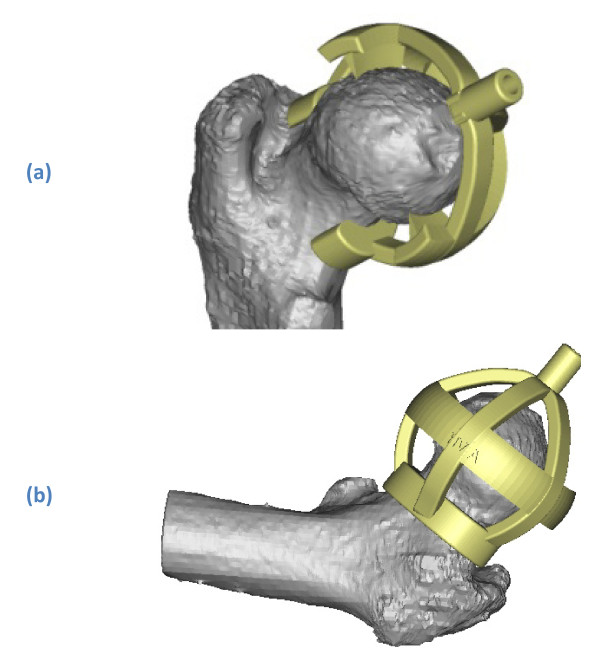
**Neck jig, designed to dril a guide wire in a pre-determined position and direction, seen from (a) medioposterior and (b) anterolateral**. [3-matic^® ^screenshot].

The three dimensional bone surface model and guide wire position are imported in the engineering design software 3-matic^® ^(Materialise NV, Leuven, Belgium) (Figure 3).

The SRA jig is designed as a hemispherical cage to fit the anterior part of the femoral head. The cage is connected to an anterior neck support. Four knifes are attached on the central arch of the cage. A drill guide cylinder is attached to the cage, reflecting the intended guide wire position. Geometrical surface offsets are applied to the jig contact geometries in accordance to previous investigation of the authors [14]. Four sharp contact struts designed to cut through remaining femoral cartilage allowing for secure bony anchorage. The neck is designed with the intention to preserve soft tissues and blood supply. As such the struts are positioned in-line and allow the rotational movement of the neck portion of the jig around the femoral neck during application. The struts offer stability in antero-posterior and in mediolateral direction. The neck portion provides additional varus-valgus stability.

The jigs are produced with a selective laser sintering (SLS) manufacturing technique, using SLS monomer, a material approved by the United States Food and Drug Administration. The parts are cleaned ultrasonically, and quality control is performed by optical scanning [Atos2 scanning device, GOM Intl. AG, Wilden, Switzerland].

### Evaluation of practical use and accuracy of positioning

Approval of the ethical committee was obtained from the Committee of Medical Ethics, University Hospitals KULeuven (nr. B32220084176). Five consecutive patients with primary osteoarthritis of the hip, scheduled for total hip arthroplasty were included. Informed consent was obtained for all patients. All five patients received a pre-operative CT scan (Somatom Sensations spiral CT, Siemens, Germany) within 6 weeks prior to surgery. The jigs were produced as described above and vapor sterilization was performed in the clinical facility according to a standard cycle for instrumentation (45 minutes at 134°C). All interventions were performed by the two orthopedic surgeons (MM/MR). A standard antero-lateral Watson-Jones approach was used. The patient was positioned supine with a pelvic tilt support under the operative side. An incision was made 2/3 proximal and 1/3 distal over the greater trochanter. The fascia lata was opened in line with the skin incision. The insertion of the gluteus medius was partially released from the greater trochanter and the anterior hip capsule was opened. The hip was dislocated. One retractor was positioned behind the femoral head and one on the femoral neck at the level of the pirifomic fossa to facilitate positioning of the neck jig. The jig was applied on the femoral head and neck with a simple rotational movement and locked in a stable snap-fit position on the anterior aspect of the femoral neck. The snap fit position and stability of the neck jig was tested qualitatively for each of the five jig designs for each patient. Qualitative feedback was provided by the surgeon. The evaluated criteria were: the uniqueness of the jig position, passive jig stability, fit from the jig with the femoral neck, knife contact, complexity during application, number of steps needed during application, and whether neck jig breakage occurred. (Table 1)

**Table 1:** Qualitative assessment

Criterion	Value range	**Surgeon's opinion for guide no**.				
		**i**	**ii**	**iii**	**iv**	**v**

**Unique position?** (number of possible positions with guide in snap-fit position, i.e. full neck contact and contact on all knifes),	1, 2, 3, ...	1	1	1	1	1
**Passive stability (snap-fit) obtained?**	Yes/No	Yes	Yes	Yes	Yes	Yes
**Full contact obtained at neck portion of jig?**	Yes/No	Yes	Yes	Yes	Yes	Yes
**All struts in contact with femoral head?**	Yes/No	Yes	Yes	Yes	Yes	Yes
**Guide failure?** (Guide breaks, or small cracks)	Yes/No	No	No	No	No	No
**Complexity to put guide in place?**	1 (not complex) to 5 (very complex)	1	1	1	2	1
**Different steps to put guide in place** (1 = single hand movement)	1, 2, 3, ...	1	1	1	1	1

**Figure 4:**
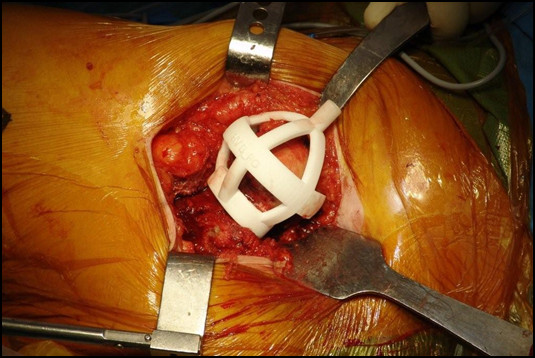
**Jig applied interaoperatively, snap-fit on proximal left femur**. Standard antero-lateral Watson Jones surgical approach for THA.

**Figure 5:**
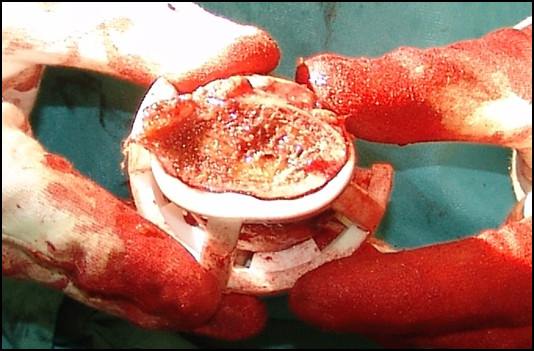
**Resected femoral head: view of cross-section of femoral neck, with jig in place**. Neck portion of the jig fits nicely on the femoral neck; and struts precisely contact the bony femoral head.

The jig was then used to drill as would have been done for a guide wire for an SRA. After positioning of the guide-wire, an osteotomy of the femoral neck was performed and the femoral head and neck were retrieved for quantitative analysis. Figure 4 shows a jig for one of the patients in snap-fit position; figure 5 represents a cross-section at the femoral neck. The further surgical intervention was carried out as per standard protocol for THA. No complications occurred during surgery. All patients were rehabilitated according to the standard protocol.

Postoperatively, a quantitative analysis of the achieved drill direction was performed. The femoral heads were optically scanned (with and without the jigs and guide wire in place) with sub-millimeter accuracy [Atos2 scanning device, GOM Intl. AG, Wilden, Switzerland] and matched onto the bone models in the preoperative planning in the Mimics software (ICP algorithm [Besl & McKay 1992]). Deviations between planned and obtained drill direction were defined as the angular deviation between the planned and optically measured drill directions, and the distance between insertion points of the planned and optically measured drill into the femoral head in the planning. Both were measured in true three dimensions.

## Results

The qualitative and quantitative evaluation results are listed in Table 1 and Table 2 respectively. In all cases the guide wire was uniquely positioned, with passive stability of the jig on the exposed proximal femur due to full contact at the neck and struts of the jig. No jigs failed during application. The complexity to put the jigs in place was low. If the device is nicely grasped, the rotational application movement is performed easily and in a single step. The quantitative benchmarking revealed maximal true 3 D deviations of 2.9 degrees and 2.1 millimeter for the drill angle and insertion point respectively. The least important angular deviation is observed in the axial plane.

**Table 2:** Quantitative assessment

Deviation measure	**Guide no**.					Overall	
	
	i	ii	iii	IV	v	min	max
Angular deviation (°)							
- true 3D	1.8	1.5	2.2	1.7	2.9	1.5	2.9
- Frontal component	0.6	1.1	1.6	1.1	1.0	0.6	1.6
- Axial component	1.5	1.0	1.0	1.0	2.4	1.0	2.4
*- Sagittal component*	*0.8*	*0.2*	*1.2*	*0.8*	*1.3*	*0.2*	*1.3*

							

Distance between insertion points (mm)	2.1	1.8	1.6	1.9	1.8	1.6	2.1

## Discussion

Literature review shows that correct patient selection and implant positioning of both femoral and acetabular components are crucial in optimizing SRA outcome. Young and active males will benefit most from SRA [2,15]. The femoral neck should be carefully prepared with a slight valgus position for the guide wire direction. Small variations on this direction may have major effect on the implant survival [10,11,13]. As reported by Davis et al [13] a 10° varus positioning will lead to a significant weakening of the femoral neck in in vitro testing of fresh frozen cadaver femora resulting in significant less resistance to stress in loading. Vail et al reported similar results for 10° of varus positioning and reported a decrease of approximately 20% in strength of the femoral neck in cases where too much valgus was given resulting in notching of the superior part of the neck^9^. It was demonstrated that a varus position of the femoral implant results in a higher complication rate [16]. Varus positioning of the femoral component and notching of the inferior femoral cortex during preparation of the neck will lead to an increased fracture risk. An exaggerated valgus position on the other hand, will however increase the risk for notching on the superior femoral cortex and thus increase the risk for femoral neck fractures [11]. Furthermore, Beaulé indicated that inaccurate femoral positioning in the saggital plane will lead to a decreased anterior femoral offset leading to impingement and a painful SRA [17]. Vail et al. [10] showed in a biomechanical study on cadaveric femurs, that small deviations in anatomic alignment of the femoral component result in marked localized increase in loading of the femoral neck.

The optimal size and position of the femoral SRA implant component can be defined before surgery with pre-operative templating. The optimal component position is either based on radiographs or on full three dimensional reconstructed images from a CT and/or MRI scanner. Reproducing the templated optimal femoral implant position during surgery relies on guide wire positioning devices in combination with visual inspection and expertise of the surgeon. The use of standard guide pin positioning devices, used either with or without navigation, has been widely explored and documented for the specific application of SRA [18].

It might be questioned whether standardized guiding instrumentation is able to reproduce the optimal alignment direction for a femoral component. A major part of the available instrumentation merely defines a limited number of drill directions. The patient anatomy defines the standard drilling orientation. The standard instrumentation does not allow to transfer the intended guide wire position as defined with pre-operative templating.

Several standard positioning devices are used. Clamping devices for example rely completely on the anatomy of the femoral neck for positioning and guide stability and only a limited number of drill holes are available for pin positioning. Other standard instrumentation follows the approach of initial instrument anchorage in the femoral head, followed by alignment of an adjustable drill guide component on the instrument to a visual reference such as the lower extremity. With these no concrete physical guidance to the optimal alignment is obtained. However, variation of alignment is possible. This is illustrated by the typical learning curve for SRA as described by Witjes et al. [19]. Positioning of the components appears to be less accurate in the beginning of the learning curve. Cobb et al illustrated that the use of CT based computer navigation in resurfacing cam-type femoral heads can increase accuracy of component positioning during the learning curve of the surgeon [20]. The use of Computer Assisted Surgery (CAS) reduces the standard deviation of implant positioning and improves repeatability independent on the surgeons experience as compared to the use of manual positioning devices [21].

Computer Assisted Surgery can be used to transfer a template surgical plan into surgery to improve accuracy of positioning of the femoral component. It significantly increases the accuracy of femoral implant positioning and facilitates positioning in a slight valgus position [22,23]. However, CAS requires bulky machinery. Pitto et al. reviewed the accuracy of CAS for femoral positioning in hips with abnormal anatomy and found that the accuracy of CAS decreased in hips with abnormal anatomy compared to normal hips [24].

The personalized custom made jig is based on three dimensional real anatomical information of CT scan, as can only be provided by CT and/or MRI medical imaging techniques [25]. The geometrical accuracy of three dimensional bone models retrieved from clinical CT scans is very high when appropriate segmentation tools and parameter values are used [14].

In the 5 cases the use of the custom made neck jig was tested with special attention for the possibility to reproduce the pre-operatively planned position of a drill during surgery. Also, the practical aspects of the use of the neck jig; in an antero-lateral approach of the proximal femur for SRA was assessed.

Data acquisition by CT scan allowed reconstructing a virtual three dimensional model which was used as template to determine the position of the drill guide. The outlining of the cartilage on the femoral head is difficult to determine on CT scan. Furthermore, due to osteoarthritis the cartilage will often be damaged. The design of the neck jig with struts with sharp edges to cut into the cartilage allows obtaining bony contact by cutting in the cartilage if present. Based on the initial positive findings the customized neck jig will be further evaluated in clinical practice.

Jig technology has proven to be reliable and accurate in guiding a drill for positioning dental implants, pedicle screw insertion, positioning long and small bone osteotomy planes and pin placement for knee arthroplasty surgery [26]. The use of personalized jigs for positioning of femoral SRA component is comparable to these indications. In each of them the jig is used to position a guide-wire for further implant positioning.

The first evaluation was performed in five patients using a jig to fit to the anterior side of the femoral neck. Our jig is designed only for application on the anterior aspect of the femoral neck. It can be used in different techniques that approach the femoral neck on the anterior side. The system can be used with an antero-lateral Watson Jones approach but also with a true anterior Smith Peterson approach. Approaching the femoral neck from the anterior side preserves the vessels in the posterior capsule of the hip. Damaging these vessels by using a posterior approach can cause avascular necrosis of the femoral neck resulting in femoral neck fractures [27,28]. Further studies should be performed to determine accuracy of guide wire positioning on cadaveric femora.

## Conclusion

This paper presented a new jig design for the specific application of guide-wire positioning for the femoral component of SRA. The first evaluation of the results from five patients using CT images and an antero-lateral surgical approach are encouraging. Accurate transfer of a three dimensional template guide wire position into surgery proved possible. The design is able to accommodate to the cartilage present on the arthritic femoral head. The custom-shaped jig allows true snap-fit stability, and the possibility to verify the correctness of the fit by visual inspection of the neck and knife contact regions with the bone. The neck jig allows for accurate and easy guide wire placement that is required for optimal resurfacing positioning as pre-operatively planned.

## List of abbreviations

SRA: Surface Replacement Arthroplasty; CAS: Computer Aided Surgery; CT: Computed Tomography; MRI: Magnetic Resonance Imaging; THA: Total Hip Arthroplasty

## Competing interests

F. Gelaude, K. De Smedt, T. Clijmans and J. Dille are employees of the Materialise Group. Materialise NV is the company that developed the neck jig and will commercialize the device.

M. Raaijmaakers and M. Mulier are orthopaedic surgeons who have no financial or other interest in this project, besides the normal clinical objective to improve surgical techniques.

## Authors' contributions

All authors read and approved the final manuscript. MR and MM: diagnosed the patients, assisted in the interpretation of the CT-scans and gave indications for the required drill orientation. They operated the patients and provided feed-back on the practical aspects of the procedure. They also supervised the interpretation of the post-operative wire position. MR and FG drafted the manuscript. MM and MR performed the final review of the manuscript. KDS, TC, JD: Performed neck jig design and production.

## Pre-publication history

The pre-publication history for this paper can be accessed here:

http://www.biomedcentral.com/1471-2474/11/161/prepub
